# Pathogenesis of Rift Valley Fever Virus Aerosol Infection in *STAT2* Knockout Hamsters

**DOI:** 10.3390/v10110651

**Published:** 2018-11-19

**Authors:** Brady T. Hickerson, Jonna B. Westover, Arnaud J. Van Wettere, Johanna D. Rigas, Jinxin Miao, Bettina L. Conrad, Neil E. Motter, Zhongde Wang, Brian B. Gowen

**Affiliations:** 1 Department of Animal, Dairy and Veterinary Sciences, Utah State University, Logan, UT 84322, USA; brady.hickerson@aggiemail.usu.edu (B.T.H.); jonna.westover@usu.edu (J.B.W.); arnaud.vanwettere@usu.edu (A.J.V.W.); jinxin.miao@aggiemail.usu.edu (J.M.); neil.motter@usu.edu (N.E.M.); zonda.wang@usu.edu (Z.W.); 2 Utah Veterinary Diagnostic Laboratory, Logan, UT 84341, USA; johanna-Rigas@idexx.com (J.D.R.); tina.conrad@usu.edu (B.L.C.); 3 IDEXX Laboratories, Mukilteo, WA 98275, USA; 4 Sino-British Research Center for Molecular Oncology, National Center for International Research in Cell and Gene Therapy, School of Basic Sciences, Academy of Medical Sciences, Zhengzhou University, Zhengzhou 450052, China

**Keywords:** Rift Valley fever virus, bunyavirus, phlebovirus, aerosol, *STAT2*, interferon

## Abstract

Rift Valley fever virus (RVFV) is an emerging pathogen capable of causing severe disease in livestock and humans and can be transmitted by multiple routes including aerosol exposure. Several animal models have been developed to gain insight into the pathogenesis associated with aerosolized RVFV infection, but work with these models is restricted to high containment biosafety level (BSL) laboratories limiting their use for antiviral and vaccine development studies. Here, we report on a new RVFV inhalation infection model in *STAT2* KO hamsters exposed to aerosolized MP-12 vaccine virus by nose-only inhalation that enables a more accurate delivery and measurement of exposure dose. RVFV was detected in hepatic and other tissues 4–5 days after challenge, consistent with virus-induced lesions in the liver, spleen and lung. Furthermore, assessment of blood chemistry and hematological parameters revealed alterations in several liver disease markers and white blood cell parameters. Our results indicate that *STAT2* KO hamsters develop a disease course that shares features of disease observed in human cases and in other animal models of RVFV aerosol exposure, supporting the use of this BSL-2 infection model for countermeasure development efforts.

## 1. Introduction

The etiologic agent of Rift Valley fever (RVF) is a tri-partite, negative-sense RNA virus (RVFV; Family *Phenuiviridae*, Genus *Phlebovirus*). Endemic to Sub-Saharan Africa and the Middle East, RVFV is a formidable pathogen able to cause severe disease in livestock and humans [1]. The majority of human RVF cases result in a biphasic, febrile illness that is typically cleared within 1 to 2 weeks. A small portion of cases progress to a severe and often fatal disease characterized by fulminant hepatitis, renal failure, retinitis, and prevalent hemorrhagic and/or encephalitic manifestations [2]. Currently, there are no licensed vaccines or therapeutics to prevent or treat RVF in humans. In addition to transmission through the bite of a mosquito, RVFV can also infect through contact with virus-containing animal excreta and tissue, or inhalation of aerosolized virus particles [3,4,5,6].

Animal models are essential for providing insights into the pathogenesis of RVFV infection. Several species including African green monkeys, marmosets, ferrets and multiple strains of mice and rats have been used to investigate the pathogenesis of RVFV when infected by the aerosol route of exposure [7,8,9,10,11,12,13,14]. These models are based on challenge with virulent strains of RVFV requiring BSL-3+ or enhanced biocontainment laboratories. In the US, RVFV is classified as a “Select Agent”, requiring stringent regulation and rigorous documentation, which in addition to the higher cost of conducting research in high containment facilities, greatly limits research with the virus. Although there are previous reports of mice deficient in type I interferon (IFN) receptors and signaling succumbing to infection by the attenuated MP-12 vaccine strain of RVFV that can be handled in BSL-2 containment [15,16], susceptibility by aerosol challenge has not been investigated.

In the present study, we exposed *STAT2* knockout (*STAT2* KO) hamsters deficient of functional STAT2 protein to aerosolized RVFV MP-12, using a delivery system in which only the snout of the animals was exposed to the aerosolized particles, and characterized the pathogenesis and natural history of disease through a temporal analysis of virologic, hematologic and serum biochemistry parameters, and histopathology. The *STAT2* KO hamsters subjected to RVFV MP-12 aerosol challenge developed many features of the human disease including acute-onset liver failure and neutrophilia, supporting the use of this new disease model for pre-clinical evaluation of promising antivirals and vaccines to protect against RVFV infection and disease.

## 2. Materials and Methods

### 2.1. Ethics Statement

All animal procedures were performed in compliance with USDA guidelines and approved by the Utah State University Institutional Animal Care and Use Committee. Studies were conducted at the AAALAC-accredited Laboratory Animal Research Center at Utah State University under protocol 2665 (approved 27 September 2016).

### 2.2. Animals

The development of the *STAT2* KO golden Syrian hamsters (*Mesocricetus auratus*) has been previously described [17]. Male and female 7–8-week-old *STAT2* KO hamsters were obtained from the breeding colony at Utah State University (Logan, UT, USA). The animals were fed autoclaved Harlan Lab Block and sterilized tap water ad libitum and acclimated for one week in the BSL-2 containment facility prior to virus challenge.

### 2.3. Virus

The molecular clone of the RVFV MP-12 vaccine strain was kindly provided by Dr. Tetsuro Ikegami (The University of Texas Medical Branch, Galveston TX, USA). The virus stock (4.0 × 10^6^ plaque-forming units (PFU)/mL; 2 passages in Vero 76 cells) was derived from a clarified cell culture lysate preparation. Virus stock was serially diluted in sterile minimum essential medium (MEM; Hyclone, Logan, UT, USA) prior to challenge to achieve target pre-nebulization concentrations. All procedures involving infectious RVFV MP-12 were performed in a class II biological safety cabinet within an animal BSL-2 laboratory. 

### 2.4. Aerosol Exposure

The infectious virus doses from aerosol exposures were determined by serially diluting virus stock and aerosolizing the various dilutions using a SCIREQ inExpose nebulization unit (SCIREQ, Montreal, QC, Canada) with a nose-only tower engineered so that only the snout of animal was exposed for 10 minutes at a constant air flow rate of 2 L/min. Aerosolized particles from each virus dilution were collected in MEM using an all-glass impinger (AGI), secured to the opening in which the snout of the hamster would be placed, allowing for collection of a representative sample for each exposure dose. Collected samples were then titrated by standard plaque assay to determine the number of PFU present in the exposure doses derived from each of the pre-nebulization virus dilutions. Results were used to calculate the average exposure dose and predicted inhaled dose as previously described [9,18,19]. The following equations were used [18]:

Exposure Dose (PFU/L) = (concentration of virus in aerosol sample collection (PFU/mL) × (volume of media in AGI (mL) − (evaporation constant (mL/min) × duration of exposure (min)))/(air flow rate (L/min) × duration of exposure (min))

Minute Volume (mL/min) = 2.1 × weight (gram)^0.75^

Inhaled Dose = minute volume (mL/min) × duration of exposure (min) × exposure dose (PFU/L)

### 2.5. Pathogenesis Study Design of Aerosolized RVFV MP-12 Infection in STAT2 KO Hamsters

**Figure 1:**
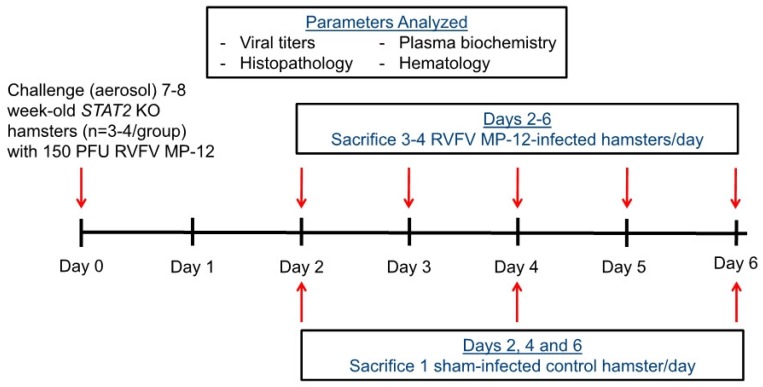
Pathogenesis study design of aerosolized Rift Valley fever virus (RVFV) MP-12 infection in *STAT2* KO hamsters.

One day prior to the start of the study, hamsters (*n* = 20) were assigned to different daily sacrifice groups by weight and sex to minimize differences across experimental groups (Figure 1). A target inhalation dose of 150 PFU was chosen for the pathogenesis study, which is about 10-fold higher than what is typically used for RVFV MP-12 hamster infections by subcutaneous or intraperitoneal challenge routes, which produce lethality within 6–7 days [20]. Groups of 6 hamsters were exposed to aerosolized virus under the same conditions and settings that were used to calculate aerosol exposure dose. The temperature during virus exposure was ambient (20–22 °C), the relative humidity was a steady state 40–60%, and the average aerosol density during exposure was 5%. Beginning on the day of challenge, animals were weighed daily and monitored for signs of disease. As shown in Figure 1, groups of hamsters were sacrificed on days 2–6 post-infection (p.i.) (*n* = 3–4 each day) and a single sham-infected control (aerosol exposure to MEM only) was sacrificed on days 2, 4 and 6 p.i. (*n* = 3 sham-infected controls). Animals were anesthetized via isoflurane inhalation to collect whole blood by retro-orbital bleed into K_3_ EDTA-coated tubes for hematologic analysis and lithium heparin-coated tubes for viral titer and plasma biochemistry analysis. Each hamster was then sacrificed and extensively perfused with phosphate-buffered saline prior to tissue collection for viral titers and histopathology. Due to an underlying health condition of unknown etiology, one of the sham-infected hamsters was removed from the analysis.

### 2.6. Quantification of Virus

Standard plaque assays for the aerosol exposure virus tests were performed similarly to previously described methods [21]. Briefly, samples from each virus dilution test were serially diluted and added to 12-well plates seeded with Vero 76 cells. Following a 1.5 h incubation, the inoculum was removed, and the cell monolayer was overlayed with agarose in MEM containing 10% FBS and 10 µg/mL gentamicin. The overlay was removed after a 5-day incubation and the monolayer stained with crystal violet. The number of PFU was determined 1 h after staining and verified 24 h later.

Viral titers in hamster serum and tissue samples were assayed using a previously described infectious cell culture assay [22]. Briefly, tissue samples were weighed and homogenized in a fixed volume of MEM, and the clarified homogenate and serum were serially titrated on Vero 76 cells. The viral cytopathic effect (CPE) was determined 10 days after plating and the 50% endpoints were calculated as previously described [23]. The lower limit of detection was 1.67 log_10_ 50% cell culture infectious doses (CCID_50_)/mL of serum and ranged from 2.83 to 4.04 log_10_ CCID_50_/g tissue.

### 2.7. Plasma Biochemistry and Hematology

To evaluate liver and kidney parameters during the course of infection, a comprehensive 18-parameter plasma biochemistry panel was performed on all plasma samples. Parameters tested include total protein (TP), albumin (ALB), alkaline phosphatase (ALP), glucose (GLU), total bilirubin (TBIL), phosphate (PHOS), cholesterol (CHOL), gamma-glutamyl transferase (GGT), alanine aminotransferase (ALT), calcium (Ca), creatinine (CRE), blood urea nitrogen (BUN), and aspartate aminotransferase (AST). The analysis was performed using a DRI-CHEM 4000 (HESKA; Des Moines, IA) according to the manufacturer’s recommendations.

For determination of complete blood counts, EDTA-anticoagulated whole-blood samples were analyzed on an automated Advia 120 Hematology Analyzer (Siemens Healthcare Diagnostic Inc., Tarrytown, NY, USA) to obtain red blood cell (RBC) counts, calculated hemoglobin (calc HGB), hematocrit (HCT), mean corpuscular volume (MCV), mean cell hemoglobin (MCH), mean corpuscular hemoglobin concentration (MCHC), cell hemoglobin concentration mean (CHCM), red cell distribution width (RDW), white blood cell (WBC) counts, band neutrophil (Band) counts, neutrophil (Neut) counts, lymphocyte (Lymph) counts, monocyte (Mono) counts, eosinophil (Eos) counts, basophil (Baso) counts, platelet (PLT) counts, and mean platelet volume (MPV).

### 2.8. Histopathology

Samples of liver, spleen, lung, kidney, intestine, brain, and heart tissues were preserved immediately in 10% neutral buffered formalin. Formalin-fixed tissues were processed and embedded in paraffin according to routine histologic techniques. Tissue sections, 5-µm thick, were stained with hematoxylin and eosin (H&E) and examined by a board-certified veterinary pathologist who was blinded to the day of sacrifice and infection status. In tissues where inflammatory lesions were present, the following semi-quantitative lesion severity scoring system was used: 0 = no lesions; no inflammatory cells or tissue necrosis present; 1 = minimal lesions; presence of few scattered inflammatory cells and/or necrotic cells (less than 10 inflammatory or necrotic cells); 2 = mild lesions; presence of small number of inflammatory cells and/or necrotic cells (less than 10 inflammatory or necrotic cells) with 1 to 2 inflammatory foci per 10× objective field; 3 = moderate lesions; presence of moderate number of inflammatory cells and/or necrotic cells (more than 10 inflammatory or necrotic cells) with 2 to 4 inflammatory foci per 10× objective field; and 4 = severe lesions; presence of numerous inflammatory cells and/or necrotic cells (more than 30 inflammatory or necrotic cells) with 5 or more inflammatory foci per 10× objective field. A minimum of 3, 10× objective fields were evaluated for each organ sample.

### 2.9. Statistical Analysis

One-way ANOVA with Dunnett’s post-test was used to correct for multiple comparisons of blood chemistry and hematology data. All statistical evaluations were done using Prism 7.0 (GraphPad Software, La Jolla, CA, USA).

## 3. Results

### 3.1. Initial Titration of RVFV MP-12 by Aerosol Challenge Route

To evaluate the susceptibility of *STAT2* KO hamsters to aerosolized RVFV MP-12, a small-scale titration was performed. Hamsters (*n* = 3/group) were challenged with varying pre-nebulization log_10_ dilutions of virus, spanning 4 orders of magnitude consisting of the same dilutions used to calculate exposure doses using an AGI as described in the methods, and observed twice per day for 14 days for morbidity or mortality. Uniform lethality was observed in all groups with the exception of 1 hamster in the group that was exposed to the lowest concentration of RVFV MP-12. Based on these results, it was determined that an exposure dose of 5700 PFU/L of virus, which calculated to approximately 150 PFU of inhaled virus, led to uniform lethality 5–7 days p.i.

### 3.2. Pathogenesis of RVFV MP-12 Aerosol Infection in STAT2 KO Hamsters

A pathogenesis study was designed to gain insights into the virology, clinical laboratory, and histopathology during the acute phase of aerosolized RVFV MP-12 infection in *STAT2* KO hamsters (Figure 1). The animals were challenged with the selected dose of approximately 150 PFU of aerosolized virus during a 10 min exposure period. Predetermined groups of hamsters (*n* = 3–4 each day) were sacrificed to assess viremia, tissue viral titers, serum biochemistry parameters, blood chemistry and histopathology on days 2–6 p.i. One hamster designated for sacrifice on day 6 p.i. expired on day 5 and was therefore included in the day 5 p.i. sacrifice group.

**Figure 2:**
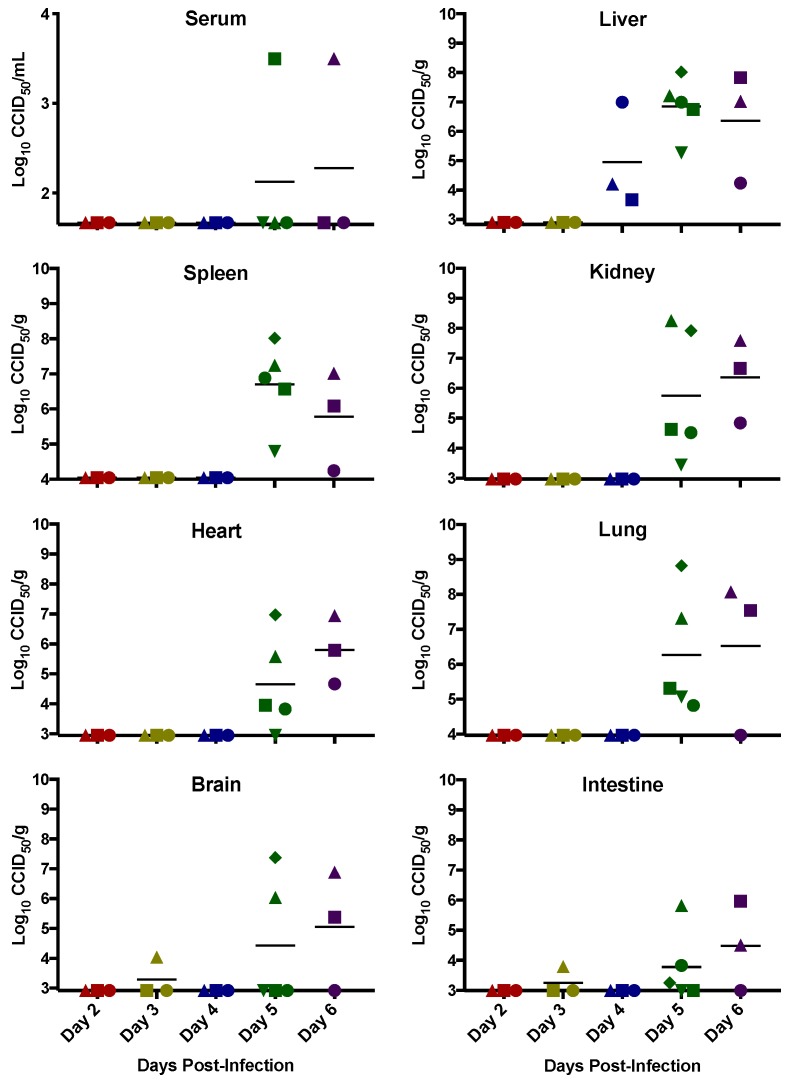
Infectious viral titers during RVFV MP-12 infection in *STAT2* KO hamsters. Groups of hamsters (*n* = 3–4) were sacrificed on the specified days post-infection for analysis of serum, liver, spleen, kidney, heart, lung, brain, and small intestine virus titers. One animal in the day 6 group succumbed to infection prior to the time of sacrifice and therefore its tissue titers (no serum could be obtained) are included in the day 5 cohort for this animal (green diamond). Unique symbols at each sacrifice day represent the same animals across all graphs.

Viral loads from the sacrificed animals are depicted in Figure 2. No virus was detected in animals on day 2 and only low levels of virus were detectable in the brain and intestine of 1 animal in the day 3 p.i. sacrifice group. The liver was a clear target for RVFV MP-12, being the first organ with consistently detectable viral loads beginning day 4 p.i. and through the end of the study. All other organs assessed had detectable levels of virus beginning day 5 p.i. and present in most animals on day 6 p.i. Despite causing a systemic infection, viremia was only detectable in two hamsters (day 5 and 6 p.i.) which were moribund at time of sacrifice and had considerable viral burdens across multiple organs. It is possible that viremia in the other hamsters was below our assay’s limit of detection but sufficient to facilitate seeding organs.

### 3.3. Alteration in Plasma Biochemistry Parameters during the Course of Infection

**Figure 3:**
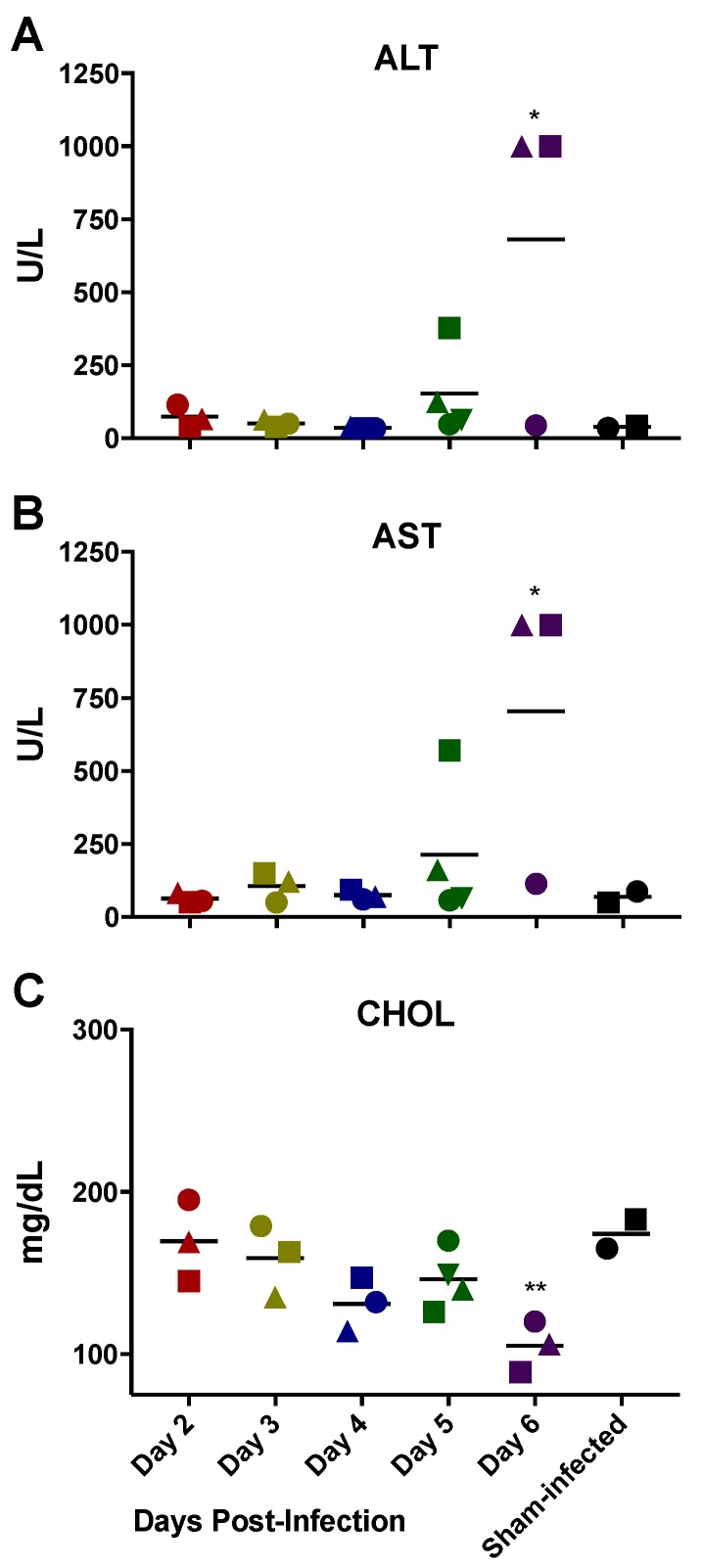
Blood chemistry values supportive of liver disease associated with RVFV MP-12 infection in *STAT2* KO hamsters. Groups of hamsters (*n* = 3–4) were sacrificed on the specified days post-infection and plasma biochemistry determined. One hamster in the day 6 group succumbed to infection prior to sacrifice and therefore could not be included in the analysis. Changes in concentrations of liver parameters (**A**) aspartate aminotransferase (AST); (**B**) alanine aminotransferase (ALT), and (**C**) cholesterol (CHOL) are shown. Unique symbols at each sacrifice day represent values for the same animals across all parameters. * *p* < 0.05 and ** *p* < 0.01 compared to day 2 RVFV MP-12-infected animals. The complete plasma biochemistry results are summarized in Supplementary Table S1.

Consistent with liver disease, ALT and AST (*p* < 0.01) were significantly elevated in the animals sacrificed on day 6 p.i (Figure 3). In addition, CHOL, a product synthesized by the liver, began to decrease on day 4 p.i. and reached significance on day 6 p.i. Also supportive of injury to the liver, a trend of decreasing GLU concentration was also observed (Supplementary Table S1).

There was no evidence of renal insufficiency in any of the RVFV MP-12-infected mice as CRE and BUN did not significantly deviate from the control animals with the exception of a single BUN value (Supplementary Table S1). In addition, urine specific gravity was highly concentrated and comparable to control animals. The complete set of plasma biochemistry parameters assessed are summarized in Supplementary Table S1.

### 3.4. Alterations of Hematologic Parameters During Infection

**Figure 4:**
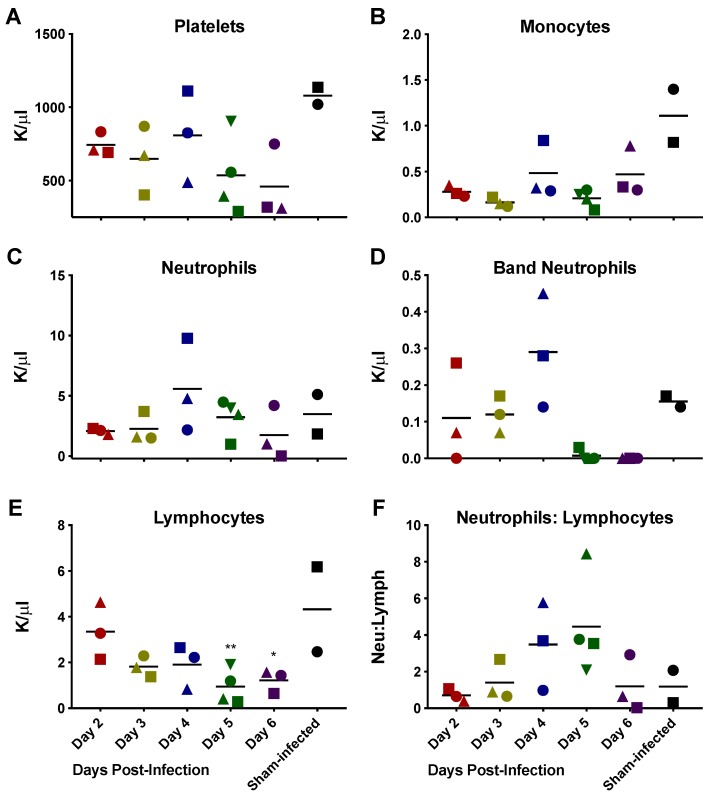
Alterations in lymphocyte and platelet parameters indicate inflammation during RVFV MP-12 infection in *STAT2* KO hamsters. Changes in (**A**) platelets; (**B**) monocytes; (**C**) neutrophils; (**D**) band (immature) neutrophils; (**E**) lymphocytes and (**F**) ratio of neutrophils to lymphocytes are shown. One hamster in the day 6 group succumbed to infection prior to sacrifice and therefore could not be included in the analysis. Unique symbols at each sacrifice day represent values for the same animal across all parameters. * *p* < 0.05 and ** *p* < 0.01 compared to day 2 RVFV-MP-12-infected animals. The complete set of hematologic parameters assessed are summarized in Supplementary Table S2.

Figure 4 shows the alterations of platelet concentration and selected WBC parameters in the *STAT2* KO hamsters during RVFV MP-12 infection. Several of the hamsters in the day 5 and 6 groups developed thrombocytopenia. The cause of the reduced platelet levels is unclear. Disruption in bone marrow function, disseminated intravascular coagulation or immune-mediated destruction are possible causes. A slight increase in the numbers of neutrophils and a decrease in the lymphocyte populations were also observed. This resulted in an inversion of neutrophil to lymphocyte numbers indicative of an inflammatory response in the hamsters, which peaked on day 5 p.i. The complete set of hematologic parameters assessed are summarized in Supplementary Table S2.

### 3.5. Histopathology

**Figure 5:**
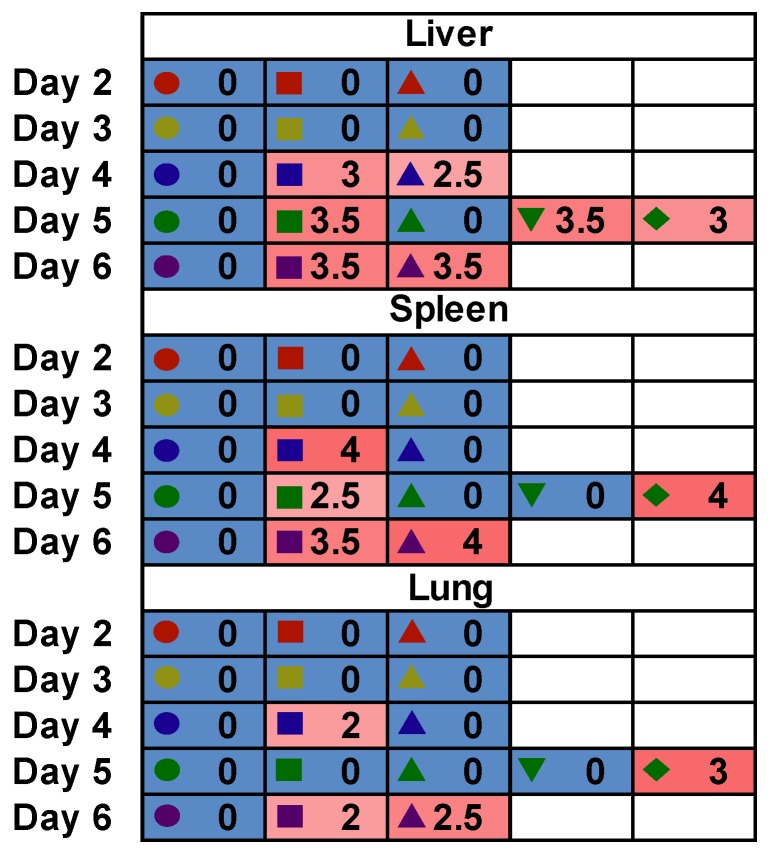
Histopathologic lesion inflammatory scores observed during the course of RVFV MP-12 infection in *STAT2* KO hamsters. Lesions were scored as follows: 0, no lesions; 1, minimal; 2, mild; 3, moderate; 4, severe. The unique symbols correspond to values for the same animal on the specified day across all evaluated parameters shown in Figure 2, Figure 3 and Figure 4. One animal in the day 6 p.i. group succumbed to infection prior to sacrifice and therefore is included in the day 5 p.i. group (green diamond).

Histologic inflammatory lesions were found in the liver, spleen and lung from day 4 to 6 p.i. and scored according to lesion severity (Figure 5). Acute multifocal neutrophilic hepatitis was observed and consisted of hepatocellular necrosis with variable numbers of neutrophils randomly distributed in the hepatic parenchyma, and none to minimal infiltration of the portal tracts with neutrophils and mononuclear inflammatory cells (likely macrophages). Lesions in the spleen consisted of a moderate to severe diffuse neutrophilic and necrotizing splenitis with lymphoid depletion and/or necrosis. Lymphoid necrosis was possibly due to direct targeting and the cytopathic effect of the virus on lymphocytes. A mild to moderate, mononuclear and neutrophilic interstitial pneumonia was present in four animals on day 4, 5 or 6 p.i. and consisted of perivascular and alveolar wall infiltration with small to moderate numbers of neutrophils and mononuclear inflammatory cells, likely a mix of macrophages and lymphocytes.

## 4. Discussion

Several models of RVFV aerosol exposure based on challenge of inbred rat strains, BALB/c mice, African green monkeys, common marmosets and rhesus macaques, have been reported [7,8,9,10,13]; however, all require animal BSL-3+ containment facilities not readily available to most researchers. For the study of viruses that require BSL-3+ containment and can have additional governmental oversight requirements (such as the US Federal Select Agent Program), the availability of BSL-2 animal models based on challenge with less pathogenic virus strains can greatly facilitate preclinical screening of promising therapeutics and vaccines, as well as investigations into viral pathogenesis. In the present study, we exposed *STAT2* KO hamsters to aerosols containing the MP-12 vaccine strain of RVFV and characterized the virology, pathology and host response associated with the infection using a nose-only exposure system facilitating more accurate delivery and measurement of virus exposure dose. Our findings indicate that *STAT2* KO hamsters are highly susceptible to inhaled RVFV MP-12 and serve as suitable hosts to model RVF-like disease contracted through aerosol exposure.

Consistent with previous work in animal models with the pathogenic ZH501 strain of RVFV [7,8,9,13,24,25,26], our study found that a variety of tissues are able to support RVFV MP-12 replication in the *STAT2* KO hamsters, demonstrating the ability of the virus to cause systemic infection and lethal disease in this model. Notably, however, previous studies with BALB/c mice, Lewis rats, and rhesus macaques challenged with aerosolized RVFV found substantial levels of viremia [9,13,26], whereas we found that only two hamsters that were moribund at the time of sacrifice, had detectable low-level viremia. Presumably, the attenuated MP-12 strain of RVFV does not replicate as efficiently or gets cleared more readily from the circulation; however, the low level of viremia appears to be sufficient to facilitate seeding of target tissues such as the liver, spleen and lung, wherein high viral loads were observed.

Despite exposure via the aerosol route, the liver was the first organ found to have measurable infectious RVFV MP-12 titers starting at 4 days p.i. Also supporting the liver as a major target organ were alterations of liver plasma parameters, and significant hepatic lesions. Based on the limited data available from human cases of RVF [1,2,27,28,29,30], and studies using other animal models for aerosol RVFV infection [9,10,14], acute hepatitis during RVFV infection is a common finding and our study suggests that *STAT2* KO hamsters develop similar inflammatory liver lesions.

## 5. Conclusions

Due to the potential of RVFV to be transmitted through aerosolization, animal models that mimic this route of exposure are valuable tools for a more complete understanding of RVF disease and pathogenesis, and the development of effective countermeasures. Further, the development of RVFV infection models compatible with BSL-2, including aerosol infection, serve to accelerate research and development in these areas. Our findings, establishing a new model of aerosol RVFV MP-12 infection in *STAT2* KO hamsters, demonstrate an infection with tropism for the liver and spleen consistent with observations in human cases and other RVF animal models. This new BSL-2 RVFV infection model will facilitate early stage drug and vaccine efficacy studies in support of new treatment and prevention strategies.

## Supplementary Materials

The following are available online at http://www.mdpi.com/1999-4915/10/11/651/s1, Table S1: Blood chemistry values during the course of the RVFV MP-12 infection in *STAT2* KO hamsters. Table S2: Hematology values during the course of the RVFV MP-12 infection in *STAT2* KO hamsters.

## References

[1] Daubney R., Hudson J. (1931). Enzootic hepatitis of rift valley fever: An undescribed virus disease of sheep, cattle, and man from east africa. J. Pathol. Bacteriol..

[2] Ikegami T., Makino S. (2011). The pathogenesis of rift valley fever. Viruses.

[3] Pepin M., Bouloy M., Bird B.H., Kemp A., Paweska J. (2010). Rift valley fever virus (bunyaviridae: Phlebovirus): An update on pathogenesis, molecular epidemiology, vectors, diagnostics and prevention. Vet. Res..

[4] Tantely L.M., Boyer S., Fontenille D. (2015). A review of mosquitoes associated with rift valley fever virus in madagascar. Am. J. Trop. Med. Hyg..

[5] Linthicum K.J., Britch S.C., Anyamba A. (2016). Rift valley fever: An emerging mosquito-borne disease. Annu. Rev. Entomol..

[6] LaBeaud A.D., Pfeil S., Muiruri S., Dahir S., Sutherland L.J., Traylor Z., Gildengorin G., Muchiri E.M., Morrill J., Peters C.J. (2015). Factors associated with severe human rift valley fever in sangailu, garissa county, kenya. PLoS Negl. Trop. Dis..

[7] Bales J.M., Powell D.S., Bethel L.M., Reed D.S., Hartman A.L. (2012). Choice of inbred rat strain impacts lethality and disease course after respiratory infection with rift valley fever virus. Front. Cell. Infect. Microbiol..

[8] Hartman A.L., Powell D.S., Bethel L.M., Caroline A.L., Schmid R.J., Oury T., Reed D.S. (2014). Aerosolized rift valley fever virus causes fatal encephalitis in african green monkeys and common marmosets. J. Virol..

[9] Reed C., Lin K., Wilhelmsen C., Friedrich B., Nalca A., Keeney A., Donnelly G., Shamblin J., Hensley L.E., Olinger G. (2013). Aerosol exposure to rift valley fever virus causes earlier and more severe neuropathology in the murine model, which has important implications for therapeutic development. PLoS Negl. Trop. Dis..

[10] Anderson G.W., Lee J.O., Anderson A.O., Powell N., Mangiafico J.A., Meadors G. (1991). Efficacy of a rift valley fever virus vaccine against an aerosol infection in rats. Vaccine.

[11] Wonderlich E.R., Caroline A.L., McMillen C.M., Walters A.W., Reed D.S., Barratt-Boyes S.M., Hartman A.L. (2017). Peripheral blood biomarkers of disease outcome in a monkey model of rift valley fever encephalitis. J. Virol..

[12] Smith D.R., Bird B.H., Lewis B., Johnston S.C., McCarthy S., Keeney A., Botto M., Donnelly G., Shamblin J., Albarino C.G. (2012). Development of a novel nonhuman primate model for rift valley fever. J. Virol..

[13] Caroline A.L., Kujawa M.R., Oury T.D., Reed D.S., Hartman A.L. (2015). Inflammatory biomarkers associated with lethal rift valley fever encephalitis in the lewis rat model. Front. Microbiol..

[14] Francis T., Magill T.P. (1935). Rift valley fever: A report of three cases of laboratory infection and the experimental transmission of the disease to ferrets. J. Exp. Med..

[15] Bouloy M., Janzen C., Vialat P., Khun H., Pavlovic J., Huerre M., Haller O. (2001). Genetic evidence for an interferon-antagonistic function of rift valley fever virus nonstructural protein nss. J. Virol..

[16] Lang Y., Henningson J., Jasperson D., Li Y., Lee J., Ma J., Li Y., Cao N., Liu H., Wilson W. (2016). Mouse model for the rift valley fever virus mp12 strain infection. Vet. Microbiol..

[17] Fan Z., Li W., Lee S.R., Meng Q., Shi B., Bunch T.D., White K.L., Kong I.K., Wang Z. (2014). Efficient gene targeting in golden syrian hamsters by the crispr/cas9 system. PLoS ONE.

[18] Guyton A.C. (1947). Measurement of the respiratory volumes of laboratory animals. Am. J. Physiol..

[19] Roy C.J., Reed D.S. (2012). Infectious disease aerobiology: Miasma incarnate. Front. Cell. Infect. Microbiol..

[20] Westover J.B., Gowen B.B. (2016). Titration of Rift Valley Fever Virus MP-12 Infection in STAT2 KO Hamsters.

[21] Baer A., Kehn-Hall K. (2014). Viral concentration determination through plaque assays: Using traditional and novel overlay systems. J. Vis. Exp..

[22] Gowen B.B., Bailey K.W., Scharton D., Vest Z., Westover J.B., Skirpstunas R., Ikegami T. (2013). Post-exposure vaccination with mp-12 lacking nss protects mice against lethal rift valley fever virus challenge. Antiviral Res..

[23] Reed L.J., Muench H. (1938). A simple method of estimating fifty percent endpoints. Am. J. Hyg..

[24] Scharton D., van Wettere A.J., Bailey K.W., Vest Z., Westover J.B., Siddharthan V., Gowen B.B. (2015). Rift valley fever virus infection in golden syrian hamsters. PLoS ONE.

[25] Smith D.R., Steele K.E., Shamblin J., Honko A., Johnson J., Reed C., Kennedy M., Chapman J.L., Hensley L.E. (2010). The pathogenesis of rift valley fever virus in the mouse model. Virology.

[26] Morrill J.C., Peters C.J. (2011). Protection of mp-12-vaccinated rhesus macaques against parenteral and aerosol challenge with virulent rift valley fever virus. J. Infect. Dis..

[27] Hartman A. (2017). Rift valley fever. Clin. Lab. Med..

[28] Laughlin L.W., Meegan J.M., Strausbaugh L.J., Morens D.M., Watten R.H. (1979). Epidemic rift valley fever in egypt: Observations of the spectrum of human illness. Trans. R. Soc. Trop. Med. Hyg..

[29] Abdel-Wahab K.S., El Baz L.M., El-Tayeb E.M., Omar H., Ossman M.A., Yasin W. (1978). Rift valley fever virus infections in egypt: Pathological and virological findings in man. Trans. R. Soc. Trop. Med. Hyg..

[30] Swanepoel R., Manning B., Watt J.A. (1979). Fatal rift valley fever of man in rhodesia. Cent. Afr. J. Med..

