# Supplementary material for: Pathogenesis of Rift Valley Fever Virus Aerosol Infection in STAT2 Knockout Hamsters
Source: Viruses. 2018 Nov 19;10(11):651. doi: 10.3390/v10110651 (PMC6265887; doi:10.3390/v10110651)
Supplement: Supplementary file 1 [file viruses-10-00651-s001.pdf]

Supplementary Materials: The following are available online at www.mdpi.com/xxx/s1, Table S1: Blood chemistry values during the course of the RVFV MP-12 infection in *STAT2* KO hamsters.

**Blood Chemistry**	Day 1	Day 2	Day 3	Day 4	Day 5	Controls
TP mg/dL	6.0 ± 0.6	5.8 ± 0.6	5.6 ± 0.7	5.8 ± 0.5	5.0 ± 0.6	5.8 ± 0.6
ALB g/dL	2.8 ± 0.2	2.8 ± 0.1	2.6 ± 0.3	2.7 ± 0.3	2.1 ± 0.5	2.7 ± 0.1
ALP U/L	207.3 ± 61.2	223.7 ± 132.6	454.7 ± 469.3	396.5 ± 284.6	721.7 ± 470.0	302.0 ± 141.4
GLU mg/dL	130.7 ± 39.11	129.3 ± 20.01	101.3 ± 22.12	74 ± 38.06	52.67 ± 57.46	132.0 ± 12.7
TBIL mg/dL	0.2 ± 0.1	0.2 ± 0.2	0.1 ± 0.0	0.1 ± 0.0	1.5 ± 1.8	0.1 ± 0.0
PHOS mg/dL	7.8 ± 1.1	8.7 ± 0.6	8.2 ± 0.8	7.9 ± 2.5	12.5 ± 3.4	9.0 ± 0.8
CHOL mg/dL	169.7 ± 25.0	159.2 ± 27.0	131.0 ± 16.5	146.3 ± 18.5	**105.0 ± 15.5****	174.0 ± 12.7
GGT U/L	10.0 ± 0.0	10.0 ± 0.0	10.0 ± 0.0	10.0 ± 0.0	10.0 ± 0.0	10.0 ± 0.0
ALT U/L	74.0 ± 38.0	51.3 ± 12.5	36.0 ± 2.7	153.5 ± 153.9	**681.3 ± 552.0***	39.5 ± 5.0
Ca mg/dL	12.8 ± 2.0	14.5 ± 0.7	13.4 ± 0.7	12.2 ± 1.1	11.8 ± 3.8	14.5 ± 0.2
CRE mg/dL	0.2 ± 0.0	0.2 ± 0.0	0.2 ± 0.0	0.2 ± 0.0	0.3 ± 0.1	0.2 ± 0.0
BUN mg/dL	17.0 ± 3.3	21.8 ± 4.2	17.8 ± 1.3	16.2 ± 3.8	36.8 ± 36.6	21.1 ± 4.4
AST U/L	63.7 ± 16.9	106.7 ± 51.0	75.0 ± 17.1	213.5 ± 242.3	**704.7 ± 511.5***	69.5 ± 26.2

Prior to infection 3-4 animals were designated for sacrifice on days 1-5 p.i. (n = 3/group for days 1-3 and 5; n = 4 for day 4). One animal in the day 5 group succumbed to infection prior to sacrifice and therefore plasma could not be obtained. Data are reported as the group mean and standard deviation (for the 2 sham-infected controls, the mean and range is given). *$P < 0.05$, **$P < 0.01$ compared to day 1 RVFV MP-12-infected animals (n = 3). Statistically significant differences are indicated by bold font.

Supplementary Materials: The following are available online at www.mdpi.com/xxx/s1, Table S2: Hematology values during the course of the RVFV MP-12 infection in *STAT2* KO hamsters.

**Hematology**	Day 1	Day 2	Day 3	Day 4	Day 5	Controls
RBC M/µl	7.9 ± 0.4	7.5 ± 0.5	7.0 ± 0.4	7.7 ± 0.2	8.4 ± 1.2	7.4 ± 0.4
Calc HGB g/dL	19.0 ± 0.8	18.2 ± 1.2	17.6 ± 1.5	19.2 ± 0.4	20.1 ± 2.5	18.0 ± 0.7
HCT %	50.4 ± 2.5	47.5 ± 2.1	44.1 ± 3.2	48.7 ± 1.4	53.8 ± 7.4	47.3 ± 2.1
MCV fL	63.4 ± 0.3	63.4 ± 1.3	63.0 ± 1.4	63.6 ± 1.2	63.9 ± 1.1	63.8 ± 0.1
MCH fL	24.0 ± 0.1	24.3 ± 0.2	25.1 ± 0.9	25.1 ± 0.7	23.8 ± 1.1	24.3 ± 0.1
MCHC g/dL	37.7 ± 1.2	38.4 ± 1.0	**39.7 ± 0.6***	39.5 ± 0.6	37.2 ± 1.0	38.1 ± 0.1
CHCM g/dL	33.5 ± 0.4	33.5 ± 0.5	33.9 ± 0.1	33.6 ± 0.5	32.6 ± 2.0	33.3 ± 0.1
RDW %	16.3 ± 0.3	16.0 ± 2.3	16.0 ± 1.1	17.3 ± 1.4	15.9 ± 2.3	17.0 ± 1.2
WBC K/µL	6.0 ± 0.9	4.5 ± 0.9	8.6 ± 4.8	4.4 ± 2.2	3.5 ± 2.5	8.74 ± 0.6
Band K/µL (%)	0.1 ± 0.1 (2.0 ± 2.7)	0.1 ± 0.1 (2.7 ± 0.6)	0.3 ± 0.2 (4.0 ± 2.6)	0.0 ± 0.0 (0.5 ± 1.0)	0.0 ± 0.0 (0.0 ± 0.0)	0.2 ± 0.0 (1.5 ± 0.7)
Neut K/µL (%)	2.1 ± 0.3 (35.7 ± 9.1)	2.3 ±1.2 (48.7 ± 16.3)	5.6 ± 3.9 (63.3 ± 16.1)	3.2 ± 1.6 (74.3 ± 8.3)	1.7 ± 2.2 (34.0 ± 34.2)	3.5 ± 2.3 (39.0 ± 24.0)
Lymph K/µL (%)	3.4 ± 1.3 (55.0 ± 13.1)	1.8 ± 0.5 (42.7 ± 15.7)	1.9 ± 1.0 (26.0 ± 17.6)	0.9 ± 0.8 (20.3 ± 8.6)	1.2 ± 0.5 (44.7 ± 20.0)	4.3 ± 2.6 (50.5 ± 33.2)
Mono K/µL (%)	0.3 ± 0.1 (6.3 ± 3.2)	0.2 ± 0.1 (3.7 ± 0.6)	0.5 ± 0.3 (5.7 ± 0.6)	0.2 ± 0.1 (5.0 ± 0.8)	0.5 ± 0.3 (20.0 ± 13.8)	1.2 ± 0.3 (9.5 ± 0.7)
Eos K/µL (%)	0.2 ± 0.1 (2.7 ± 2.1)	0.1 ± 0.0 (2.3 ± 1.2)	0.1 ± 0.1 (0.3 ± 0.6)	**0.0 ± 0.0* (0.0 ± 0.0*)**	0.0 ± 0.0 (0.7 ± 0.6)	0.5 ± 0.7 (0.5 ± 0.7)
Baso K/µL (%)	0.0 ± 0.0 (2.0 ± 1.0)	0.0 ± 0.0 (0.0 ± 0.0)	0.1 ± 0.1 (0.7 ± 1.2)	0.0 ± 0.0 (0.0 ± 0.0)	0.0 ± 0.0 (0.7 ± 1.2)	0.0 ± 0.0 (0.0 ± 0.0)
PLT K/µL	744.3 ± 77.3	647.7 ± 234.9	808.7 ± 312.4	536.0 ± 269.0	459.3 ± 250.9	1079.0 ± 82.7

MPV fL	6.2 ± 0.1	6.2 ± 0.4	6.3 ± 0.4	6.5 ± 0.2	7.6 ± 2.1	6.3 ± 0.1

Prior to infection 3-4 animals were designated for sacrifice on days 1-5 p.i. (n = 3/group for days 1-3 and 5; n = 4 for day 4). One animal in the day 5 group succumbed to infection prior to sacrifice and therefore plasma could not be obtained. Data are reported as the group mean and standard deviation (for the 2 sham-infected controls, the mean and range is given). *$P < 0.05$, **$P < 0.01$ compared to day 1 RVFV MP-12-infected animals (n = 3). Statistically significant differences are indicated by bold font.
